# The complete chloroplast genome of *Zabelia dielsii* (Graebn.) Rehd. (Caprifoliaceae), an important horticultural plant in China

**DOI:** 10.1080/23802359.2019.1630336

**Published:** 2019-07-12

**Authors:** Yanan Cao, Tianlei Xie

**Affiliations:** a College of Plant Protection, Henan Agricultural University, Zhengzhou, China;; b College of Electrical and Mechanical Engineering, Central South University of Forestry and Technology, Changsha, China

**Keywords:** *Zabelia dielsii*, complete chloroplast genome, Caprifoliaceae

## Abstract

The genus *Zabelia* R. Br. has been an important horticultural plant in the family Caprifoliaceae. However, due to the lack of efficient molecular markers and conservation efforts in recent decades, accompanied with deforestation, the germplasm resources are under severe threat, yet the systematic position of *Zabelia* is not clearly understood. In the present study, we analyzed the complete chloroplast (cp) genome of *Zabelia dielsii* (Graebn.) Rehd., which represents the first reported case of *Zabelia*. It was 155,584 bp in length, comprising a pair of 23,434 bp inverted repeat regions (IRs) separated by a large single-copy (LSC) region (89,521 bp) and a small single-copy (SSC) region (19,062 bp). Phylogenetic analysis supported the monophyly of Caprifoliaceae and suggested that *Z. dielsii* was closely related to *Kolkwitzia amabilis* and *Dipelta floribunda*. This complete chloroplast genome will contribute to further studies on population genetics, phylogeny, and conservation biology in *Zabelia*.

The genus *Zabelia* R. Br. is among one of the most well-known flowering plants in Caprifoliaceae. However, to date, no study appears to have fully addressed the systematic position of *Zabelia* (Jacobs et al. 2010). Furthermore, deforestation in the past decades have caused severe reduction of wild populations and imposed threats to germplasm resources. Hence, it is quite urgent to develop a large number of variable markers that can help in future studies on phylogeny and conservation in *Zabelia*.

In this study, the complete and annotated sequence was presented for *Z. dielsii*. Fresh leaf material was collected from the Maijishan (N 34° 20′ 41.17″, E 106° 00′ 42.32″) and the voucher specimen was stored in Henan Agricultural University Herbarium. Total genomic DNA was extracted using DNA Plantzol (Invitrogen) and sequenced on the Illumina Hiseq 2500 Platform by Beijing Genomics Institute (BGI; Shenzhen, China). For the data that BGI produced, we then filtered and assembled the complete genome using CLC genomics workbench (http://www.clcbio.com/products/clc-assembly-cell/). The genome was annotated using the DOGMA (Dual Organellar GenoMe Annotator) database (Wyman et al. 2004) and submitted to GenBank with Sequin (http://www.ncbi.nlm.nih.gov/). Start/stop codons and intron/exon boundaries were identified with that of the cp genome of *Dipelta floribunda* (KP718628) as reference using MAFFT (Katoh and Standley 2013). We also used tRNAscanSE (Schattner et al. 2005) to verify the tRNA boundaries determined by DOGMA with default settings. Then, we used OrganellarGenome DRAW (http://ogdraw.mpimp-golm.mpg.de/) (Lohse et al. 2013) to draw the physical map of the cp genome of *Z. dielsii*. Finally, to confirm the phylogenetic position of *Z. dielsii*, a phylogenetic tree was conducted on 18 Caprifoliaceae species (Figure 1) using one species each for *Sinadoxa corydalifolia*, *Tetradoxa omeiensis* and *Adoxa moschatellina* as outgroups based on previous studies (Jacobs et al. 2011) with RAxML (Stamatakis 2014).

**Figure 1.**
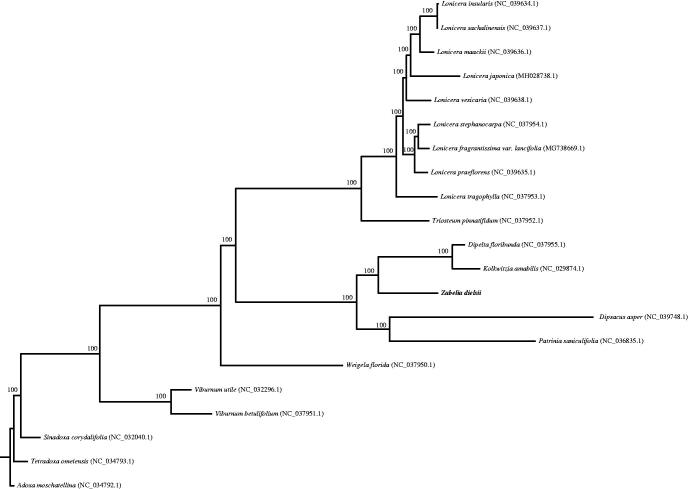
Phylogenetic tree based on 21 complete chloroplast genome sequences from the order Dipsacales.

The full length of the cp genome of *Z. dielsii* was 155,584 bp with 61.6% AT content (GenBank accession number: MK543511). Its quadripartite structure was composed of a LSC region (89,521 bp), a SSC region (19,062 bp), and a pair of inverted repeated regions (IRs; 23,434 bp). The genome encoded a set of 129 genes, including 83 protein-encoding genes with one pseudogene (*accD*), 38 tRNA genes, and 8 rRNA genes. It is noteworthy that 15 genes were totally duplicated in the IR regions. The genome organization, gene content, and the relative positions of the 129 genes of *Z. dielsii* were almost identical to those of *Kolkwitzia amabilis* (Bai et al. 2017).

Phylogenetic analysis showed all the species of Caprifoliaceae formed a monophyletic clade with 100% bootstrap value and *Z. dielsii* was closely related to *K. amabilis* and *D. floribunda* (Figure 1), as in earlier phylogenetic studies (Jacobs et al. 2010; Wang et al. 2015). The newly characterized complete cp genome of *Z. dielsii* will provide essential genetic information to develop DNA markers for its wild populations and contribute to further studies on population genetics and conservation biology in the future.
